# Identification of vitamin D and other bone metabolism parameters as risk factors for primary bone marrow oedema syndrome

**DOI:** 10.1186/s12891-018-2379-x

**Published:** 2018-12-22

**Authors:** Nicola Oehler, Haider Mussawy, Tobias Schmidt, Tim Rolvien, Florian Barvencik

**Affiliations:** 1 Department of Orthopaedic Surgery, University Medical Center Hamburg-Eppendorf, Martinistraße 52, 20246 Hamburg, Germany; 2 Department of Osteology and Biomechanics, University Medical Center Hamburg-Eppendorf, Lottestraße 59, 22529 Hamburg, Germany

**Keywords:** Bone marrow oedema syndrome, BMES, Vitamin D, Bone metabolism

## Abstract

**Background:**

The aetiology and pathogenesis of primary bone marrow oedema syndrome (BMES) remain unclear. This retrospective cross-sectional study in a large cohort of patients with BMES was performed to characterise the overall skeletal status and turnover in patients with BMES, with the aim of identifying risk factors for this disease.

**Methods:**

Patients who were diagnosed with BMES on the basis of clinical and radiological (magnetic resonance imaging) findings in our outpatient clinic were identified retrospectively. Patient history, co-existing metabolic disorders, bone metabolism parameters (serum calcium, phosphate, 25-OH-D_3_, bone-specific alkaline phosphatase, parathyroid hormone, and osteocalcin, and urinary deoxypyridinoline) and bone mineral density (as measured by dual-energy X-ray absorptiometry) were extracted from the medical records. Patients with secondary causes for BMES were excluded from the study.

**Results:**

Of the 171 patients, 65 were identified without secondary cause for BMES. Of the 65 patients, 61.5% were female. The mean age was 49.5 ± 16.7 years, and age-related BMES prevalence showed two peaks, one in adolescence (11–20 years) and one at an older age (51–70 years). BMES predominantly affected the weight-bearing joints, namely, the ankle/foot (55.1%), knee (22.4%) and proximal femur (16.3%). Thyroid disorders and secondary hyperparathyroidism were highly prevalent (21.5 and 21.4%, respectively). On average, the cohort had elevated deoxypyridinoline levels and low 25-OH-D_3_ levels (19.0 ± 7.5 μg/l in patients without vitamin D supplementation). Osteopenia and osteoporosis were diagnosed in 47.4 and 17.5% of patients, respectively.

**Conclusions:**

BMES is associated with high bone turnover. Patients who are diagnosed with BMES should be screened carefully for bone metabolism disorders and their potential risk factors.

**Electronic supplementary material:**

The online version of this article (10.1186/s12891-018-2379-x) contains supplementary material, which is available to authorized users.

## Background

Bone marrow oedema (also known as bone marrow lesion) is characterised by the excessive accumulation of fluid within the bone that causes nonspecific pain and high-signal-intensity alterations on magnetic resonance fluid-sensitive sequences [1]. A number of these lesions are secondary to various pathologies, including trauma, osteoarthritis, inflammatory arthritis, benign and malignant neoplasms, infections and metabolic disorders [2, 3]. However, primary bone marrow oedema lesions that lack an obvious cause also exist. These lesions are most commonly found in the bones around the hip, knee, ankle and foot and have been variously termed regional migratory osteoporosis, transient osteoporosis, and, most recently, bone marrow oedema syndrome (BMES) [4, 5]. The multiple names, and the fact that it is a diagnosis by exclusion, reflect the prevailing uncertainty about the aetiology of BMES [4]. However, several hypotheses about BMES pathogenesis have been proposed. One suggests that it is caused by nerve compression, another that it is the result of increased intramedullary pressure due to altered venous outflow [6]. However, more recently, clinical and histological studies on bone metabolism in BMES suggested that deterioration of bone mineralisation due to vitamin D deficiency may contribute to BMES [7–10]. However, these studies involved small patient cohorts and/or only assessed a few diagnostic aspects such as bone mineral density (BMD) or vitamin D deficiency. Thus, the aetiology and pathogenesis of BMES remain poorly understood.

To improve our understanding of why and how BMES develops, we conducted a retrospective study with a large cohort of patients with BMES in which we assessed the relationship between a wide variety of bone metabolism, bone quality, and potential risk factors and BMES.

## Methods

### Patient selection

The cohort consisted of 171 patients who were diagnosed with BMES at our specialized osteologic outpatient clinic. Patients with clinical, radiological or laboratory evidence of avascular necrosis *(n = 11)*, infection *(n = 4)*, tumours *(n = 3)*, trauma/elevated mechanical stress *(n = 58)*, arthrosis *(n = 16)*, pregnancy *(n = 5)*, anorexia nervosa *(n = 6)*, or complex regional pain syndrome *(n = 3)* were excluded from the study. In total 65 patients were identified without having causes described above for BMES. BMES was diagnosed on the basis of a history of acute non-traumatic onset of pain in the affected limb and abnormal bone marrow signal intensity on magnetic resonance imaging (MRI), namely, high signal intensity on fat-suppressed T2 weighted and short-tau inversion recovery images and low signal intensity on T1 weighted images. The MRI scans were obtained at specific local radiology institutes with varying magnets (1.5 and 3.0 Tesla) and protocols, after which they were assessed by a musculoskeletal radiologist and an experienced orthopaedic surgeon. A detailed medical history with a special focus on potential risk factors for impaired bone metabolism (age, sex, body mass index, nicotine abuse, medication, pre-existing diseases and history of fractures) was obtained from all patients.

### Laboratory assessments

The patients underwent biochemical analyses of bone metabolism markers, including the serum levels of calcium, phosphate, 25-OH-D_3_, bone-specific alkaline phosphatase (BAP), parathyroid hormone (PTH), and osteocalcin, and the urinary level of deoxypyridinoline (DPD). The analyses were performed in a Department of Clinical Chemistry. The reference values of each variable are those of the local laboratory (Table 1). Vitamin D insufficiency and deficiency were defined as 25-OH-D_3_ levels of 20–30 μg/l and < 20 μg/l, respectively. These values reflect those of widely accepted thresholds [11]. Patients were diagnosed with secondary hyperparathyroidism when their PTH levels exceeded 84 ng/l, their calcium serum levels were within the (low) normal range and their 25-OH-D_3_ levels were reduced. Patients with permanent proton pump inhibitor (PPI) intake, calcium serum levels below 2.20 mmol/l and elevated gastrin levels (> 115 ng/l) were diagnosed with PPI-induced hypochlorhydria.

**Table 1:** Clinical characteristics and laboratory and dual-energy X-ray absorptiometry values at the time of bone marrow oedema syndrome diagnosis

Diagnostic characteristics	*n* (%) or mean ± SD	
Patient history and co-existing diseases		
Nicotine abuse	16/56 (29.6)	
BMI, kg/m^2^, *n* = 65	24.2 ± 4.7	
Thyroid disorders	14/65 (21.5)	
Long-term^a^ PPI medication	11/62 (17.7)	
Long-term^a^ corticosteroid therapy	7/58 (11.9)	
Serum and urine osteological laboratory values, *n* = 56		Reference values
Phosphate, mmol/l	0.97 ± 0.18	0.77–1.50
Calcium, mmol/l	2.26 ± 0.10	2.13–2.63
25-OH-D_3_, μg/l	22.9 ± 10.3	> 30.0
> 30.025-OH-D_3_, μg/l, unsupplemented patients, *n* = 36	19.0 ± 7.5	> 30.0
PTH, ng/l	61.2 ± 31.5	17.0–84.0
DPD, nmol/mmol	7.1 ± 4.1	3.0–7.0
Bone-AP, μg/l	14.8 ± 11.2	5.2–24.4
Osteocalcin, μg/l	20.6 ± 12.9	5.4–59.1
Bone metabolism disorders		
*Supplemented group, n = 20*		
Sufficient (25-OH-D_3_ ≥ 30 μg/l)	11 (55)	
Insufficient (25-OH-D_3_ 20 to < 30 μg/l)	5 (25.0)	
Deficient (25-OH-D_3_ 10 to < 20 μg/l)	4 (20.0)	
Very deficient (25-OH-D_3_ < 10 μg/l)	0 (0.0)	
*Unsupplemented group, n = 36*		
Sufficient (25-OH-D_3_ ≥ 30 μg/l)	3 (8.3)	
Insufficient (25-OH-D_3_ 20 to < 30 μg/l)	12 (33.3)	
Deficient (25-OH-D_3_ 10 to < 20 μg/l)	21 (58.3)	
Very deficient (25-OH-D_3_ < 10 μg/l)	3 (8.3)	
Secondary hyperparathyroidism, *n* = 56	12 (21.4)	
Primary hyperparathyroidism, *n* = 56	1 (1.8)	
PPI-induced hypochlorhydria, *n* = 56	3 (5.4)	
Osteopenia (lumbar spine T-score ≤ −1.0 and > −2.5), *n* = 57	27 (47.4)	
Osteoporosis (lumbar spine T-score ≤ −2.5), *n* = 57	10 (17.5)	
Bone mineral density, *n* = 57		
Z-score LWS	−1.1 ± 1.4	
Z-score left femur	−0.8 ± 1.0	
Z-score right femur	−0.7 ± 0.9	

^a^ At least a year

*Bone-AP* bone-alkaline phosphatase, *BMI* body mass index, *25-OH-D*_*3*_ 25-hydroxy vitamin D, *DPD* deoxypyridinoline, *PPI* proton pump inhibitor, *PTH* parathyroid hormone, *SD* standard deviation

### Dual-energy X-ray absorptiometry

In 57 patients, BMD at the lumbar spine and both proximal femurs (if indicated) was measured by dual-energy X-ray absorptiometry (DXA; Lunar iDXA, GE Healthcare; Madison, WI, USA) according to good clinical practice guidelines. The scans were performed at the same time as the medical history and laboratory data were obtained. The patients were placed in the supine position and scanned according to the manual supplied by the manufacturer. The BMD of the projected bone area was expressed as grams per square centimetre (g/cm^2^), and the corresponding T- and Z-scores were calculated. The T-score was used to determine if the patient had normal BMD (> − 1.0), osteopenia (T-Score between − 1.0 and − 2.5) or osteoporosis (≤ − 2.5) on the basis of the World Health Organization criteria for osteoporosis.

### High-resolution peripheral quantitative computed tomography

In 37 of the patients, the bone microstructure of the non-dominant distal radius and contralateral distal tibia was assessed by high-resolution peripheral quantitative computed tomography (HR-pQCT; XtremeCT, SCANCO Medical, Brüttisellen, Switzerland) if the patients had an elevated fracture risk and/or degenerative lumbar alterations which falsify aBMD measurement at the spine. The HR-pQCT scans were performed at the same time as the medical history and laboratory data were obtained and the DXA scans were performed. The default in vivo settings were used, namely, 60 kVp, 1000 μA, 100 ms integration time and voxel size of 82 μm as described previously [12]. These settings generate 3-dimensional microstructural data of the cortical and trabecular compartments. The manufacturer’s standard protocol was used to generate various bone microstructure variables, namely, bone volume to total volume ratio, trabecular number, trabecular thickness, and cortical volumetric BMD. For further interpretation our data were expressed as percentage of recently published age-, and sex- specific reference values by Burt et al. [13].

### Statistical analysis

All statistical analyses were performed using GraphPad Prism 7 software. All continuous variables were expressed as mean ± standard deviation (SD). Groups were compared in terms of continuous variables using Student’s *t*-test and in terms of categorical variables using the Chi-squared test.

## Results

### Age at diagnosis and localisation of BMES lesions

**Fig. 1:**
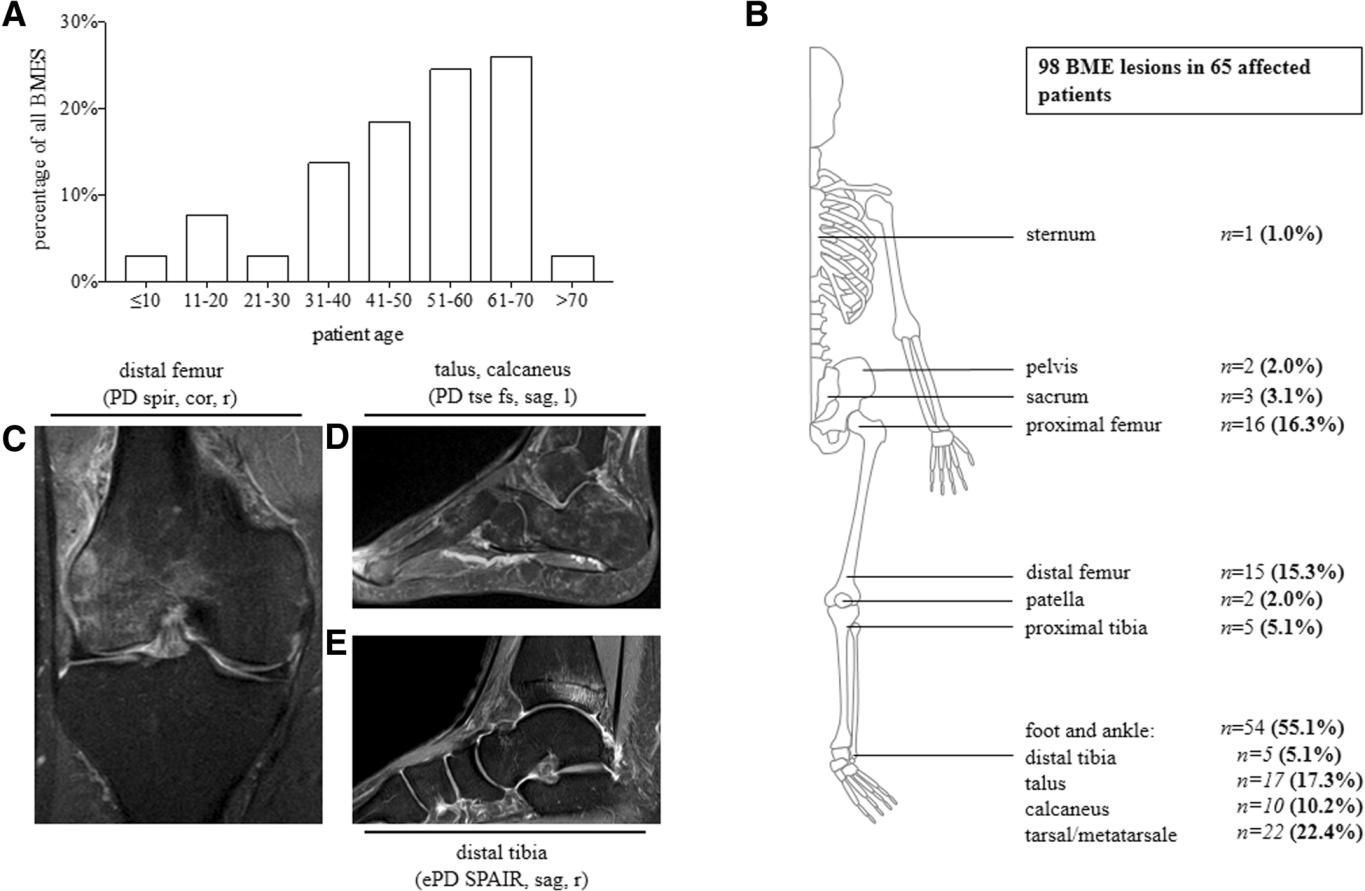
Age distribution of the 65 patients with primary bone marrow oedema syndrome (BMES) and localisation of the 98 lesions. **a** Frequency of patients in specific age brackets. **b** Anatomical location of the lesions. The data are shown as number (%). **c–e** Magnetic resonance imaging (MRI) scans of BMES lesions in the knee, distal tibia and tarsal bones. The scans show the distinctive MRI patterns. PD, proton density; SPIR, spectral presaturation with inversion recovery; SPAIR, spectral attenuated inversion recovery; tse, turbo spin echo; fs, fat suppressed; cor, coronal; sag, sagittal; r, right; l, left

Of the 171 patients, 65 patients with primary BMES were included in this study. Of these, 61.5% were female and the mean age was 49.5 ± 16.7 (range, 10–83) years. Interestingly, the age at BMES diagnosis exhibited a bimodal distribution. Thus, there was a small peak at the age of 11–20 years (7.7% of all patients) that was followed by a trough at 21–30 years. Thereafter, the prevalence of BMES in the cohort started rising again and eventually peaked at 51–70 years of age (50.7% of all patients) (Fig. 1**a**).

27 of the patients (41.5%) had more than one BMES lesion in either the same or different joints. Thus, 98 BMES lesions were detected in total. The foot and ankle were most commonly affected (*n* = 54 lesions, 55.1% of all lesions), followed by the knee (*n* = 22, 22.4%) and the proximal femur (*n* = 16, 16.3%). Five lesions (5.1%) were found in the pelvis/sacrum and there was one lesion in the sternal bone. Lesions in the upper extremities were not found. The overall skeletal distribution of the BME can be found in Fig. 1b. Figure 1c–e shows examples of the BMES lesions on the distal femur, distal tibia and tarsal bones.

### Patient history and concomitant diseases

The medical records indicated that 29.6% of the patients were abusing nicotine, 17.7% reported long-standing daily intake of PPI and 11.9% were undergoing long-term systemic corticosteroid therapy at diagnosis. Interestingly, 14 (21.5%) of the 65 patients had previously diagnosed thyroid disorders (13 had hypothyroidism and one had hyperthyroidism) and most of these patients (10/14) received daily thyroid hormone therapy. Onset of disease showed a peak during winter time (Additional file 1: Figure S1).

### Bone turnover variables and prevalence of bone metabolism disorders

**Fig. 2:**
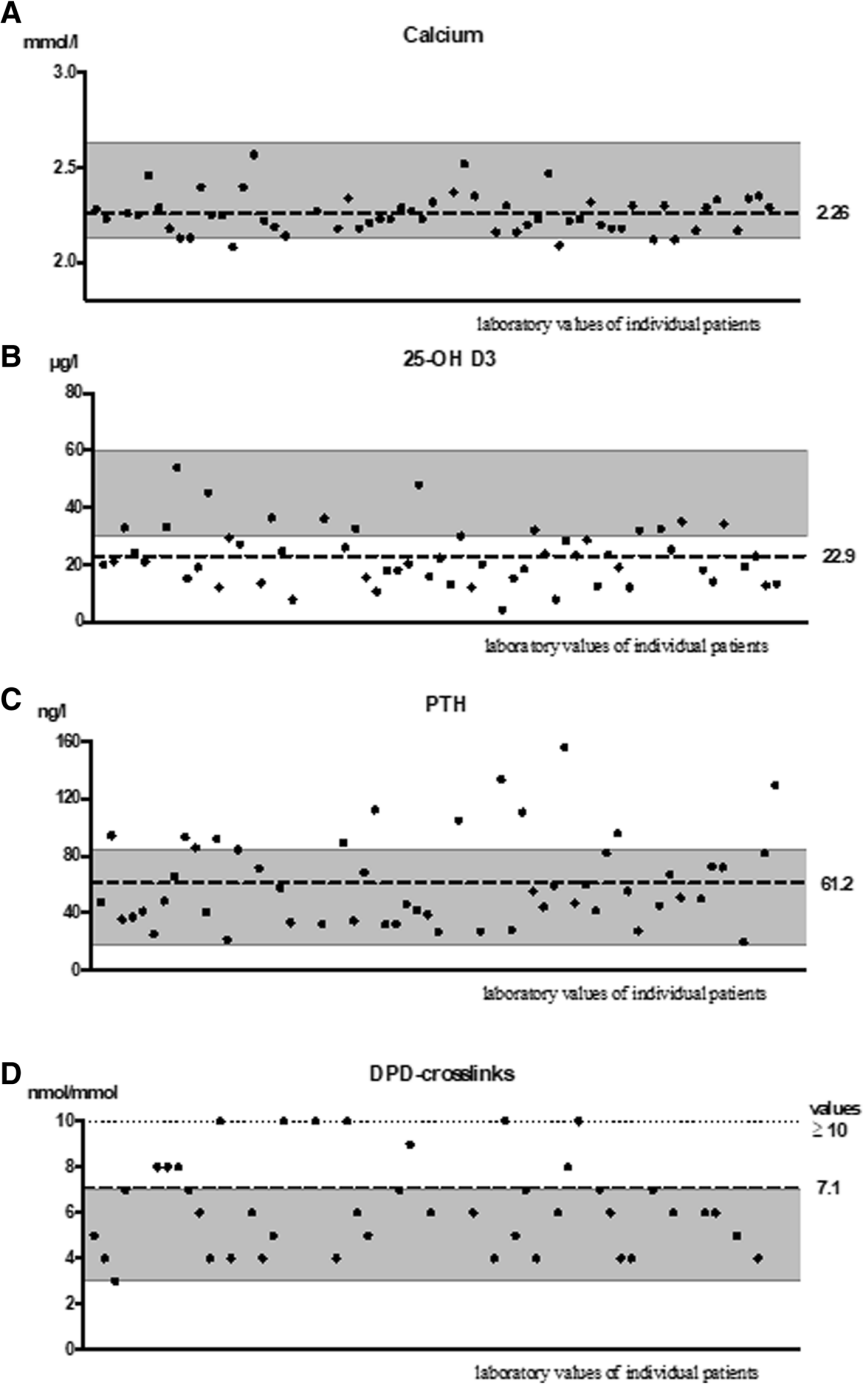
Bone turnover parameter of the patients with bone marrow oedema syndrome (BMES). **a** Serum calcium levels (*n* = 56; reference, 2.13–2.63 mmol/l). **b** Serum 25-OH-D_3_ levels (*n* = 56; reference, 30–60 μg/l). **c** Serum parathyroid hormone (PTH) levels (*n* = 56; reference, 17–84 g/l). **d** Serum desoxypyridinoline (DPD) levels (*n* = 56; reference, 3–7 nmol/mmol). The dots indicate the values of each patient. The dashed line indicates the mean of the cohort. The grey-shaded area indicates the reference values of the local laboratory for each variable

The mean serum phosphate level was 0.97 ± 0.18 mmol/l. While only four patients (7.1%) had hypocalcaemia, the mean calcium serum level of the cohort was 2.26 ± 0.10 mmol/l, which is near the lower limit of the reference range (2.13 mmol/l) (Table 1 and Fig. 2a). The laboratory tests revealed that three patients (5.4%) demonstrated hypochlorhydria due to long-standing PPI intake (Table 1).

The mean 25-OH-D_3_ level in the 56 patients for whom these data were available was 22.9 ± 10.3 μg/l: this value lies below the optimal level of > 30 μg/l [14] (Table 1 and Fig. 2b). However, this may not reflect the usual 25-OH-D_3_ levels in the cohort patients because 20 (35.7%) of our patients had recently started taking vitamin D supplements on the advice of local orthopaedists. Mean level of 25-OH-D_3_ of those 20 patients (35.7%) who had recently started taking vitamin D supplementation was 30.1 ± 10.8 μg/l. Indeed, the 36 patients who had not been treated recently with vitamin D supplements had a mean 25-OH-D_3_ level of 19.0 ± 7.5 μg/l. Of these patients, 12 (33.3%) had insufficient 25-OH-D_3_ levels (20–30 μg/l) and 21 (58.3%) had 25-OH-D_3_ deficiency (< 20 μg/l). Thus, only three (8.3%) of the unsupplemented patients had adequate 25-OH-D_3_ levels (by contrast, 55% of the supplemented patients had adequate 25-OH-D_3_ levels) (Table 1).

The mean serum PTH levels were 61.2 ± 31.5 ng/l, which is near the upper limit of the normal range (84.0 ng/l). Indeed, 13 patients (23.2%) were diagnosed with hyperparathyroidism on the basis of the laboratory tests. Of these, one had primary hyperparathyroidism and the remaining 12 were diagnosed with secondary elevation of PTH levels (Table 1 and Fig. 2c).

DPD-crosslinks are a biochemical marker of bone resorption. The DPD-crosslink levels were elevated in 11 patients (19.6%) and in the cohort: the mean value was 7.1 ± 4.1 nmol/mmol (upper limit, 7.0 nmol/mmol) (Table 1 and Fig. 2d). However, the cohort had normal levels of BAP and osteocalcin, which are markers of bone formation (14.8 ± 11.2 μg/l and 20.6 ± 12.9 mg/l, respectively).

### BMD, as measured by DXA, and bone microstructure, as measured by HR-pQCT

Of the 65 patients, 57 underwent measurement of bone mineral density by DXA in our institution. This cohort had mean Z-scores of − 1.1 ± 1.4 at the lumbar spine and − 0.8 ± 1.0 and − 0.7 ± 0.9 at the left and right femur, respectively. The mean T-scores of the adult individuals of the cohort were − 1.2 ± 1.6 for the lumbar spine, − 1.3 ± 1.2 for the left femur and − 1.2 ± 1.2 for the right femur. As a result of the T-scores, 27 patients (47.4%) were diagnosed with osteopenia and 10 (17.5%) were found to have osteoporosis (Table 1).

**Fig. 3:**
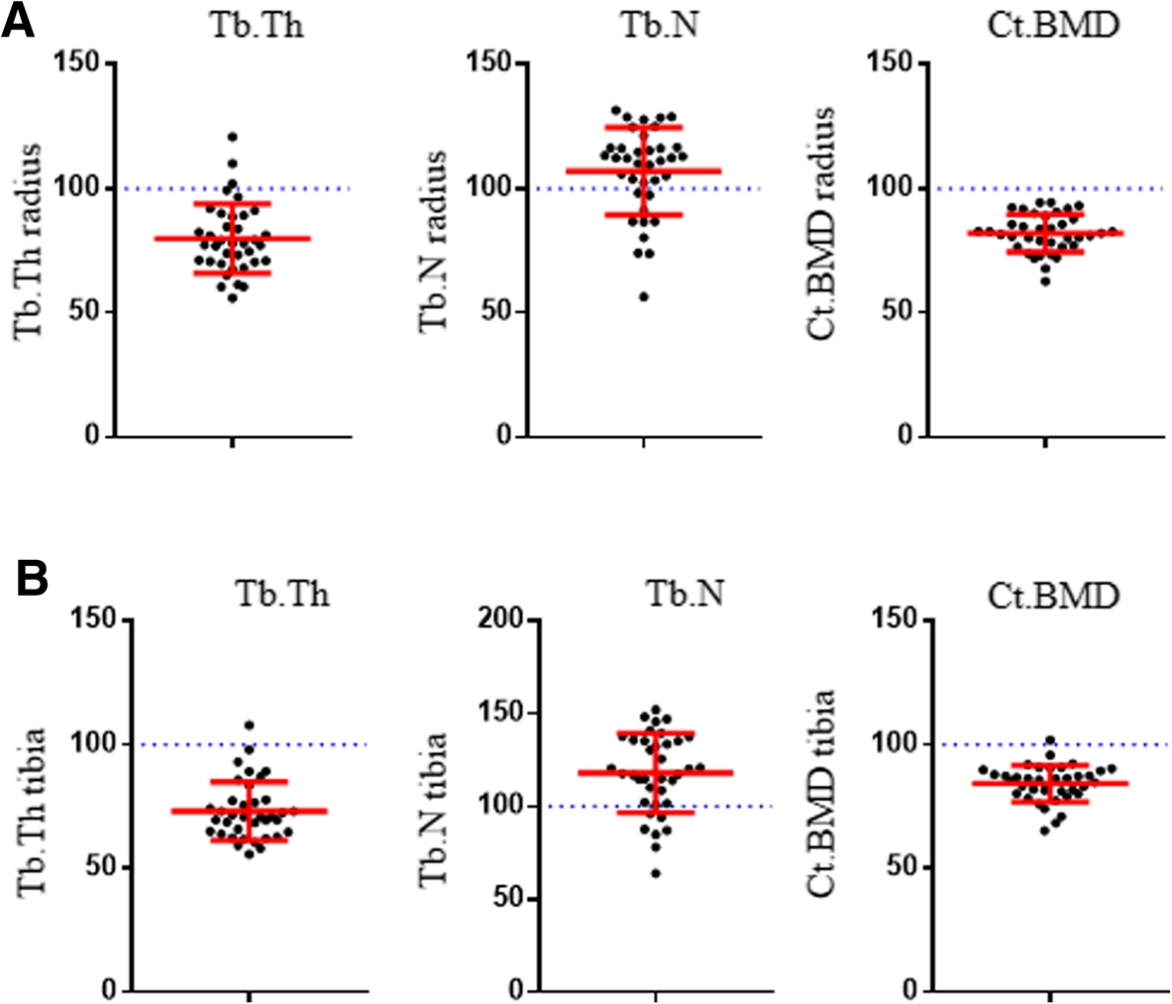
Bone structure at the radius (**a**) and tibia (**b**) of patients with bone marrow oedema syndrome (BMES) assessed by HR-pQCT. Trabecular thickness (Tb.Th.), trabecular number (Tb.N.), and cortical volumetric BMD (Ct.BMD) of 37 patients were compared to age- and sex-specific reference values provided by Burt et al.

We also assessed the bone quality of 37 patients using HR-pQCT. The trabecular values and cortical bone mineral density were compared to the age- and sex-specific reference curves provided by Burt et al. in 2016 [13]. The data showed reduced trabecular thickness and cortical volumetric BMD with normal to high trabecular number at the distal radius and distal tibia in our patient cohort (Fig. 3). The alterations in bone structure were similar between the upper and lower extremities in our patient cohort despite the fact that BMES affected almost the lower limb.

## Discussion

The current study showed that, in patients with newly diagnosed BMES, age at onset exhibited two peaks, one in adolescents (11–20 years of age) and one in older/elderly patients (51–70 years of age). The majority (61.5%) were female, a fifth had thyroid disorders (21.5%), another fifth (21.4%) had secondary hyperparathyroidism and another fifth (19.6%) had elevated DPD levels, which indicate increased bone resorption. In addition, the vast majority (91.7%) of the patients who had not recently undergone vitamin D supplementation exhibited vitamin D insufficiency or deficiency. A peak of disease onset was seen during the winter time were vitamin D levels are generally lower than in summer months. Furthermore, the patients exhibited alterations in trabecular and cortical bone structure.

Numerous studies have assessed the disease characteristics of BMES. They showed that male patients predominate and the vast majority of lesions are found on the lower extremity (98%), with the proximal femur being the most common site, followed by the knee and the ankle/foot [3, 4, 6, 15–19]. By contrast, our patient cohort showed a female predominance. However, we did find, like the other studies, that the BMES lesions in our cohort showed a strong predilection for the lower weight-bearing extremities (99%). This suggests that external load is a predisposing factor for BMES [4]. However, unlike previous observations, the lesions in our patients were markedly concentrated in the foot and ankle region, followed by the knee and then the proximal femur. This may reflect the fact that the other studies focused on one selected joint region, and included subchondral fractures (43% in their study group) at the hip [15] or including subchondral fractures (11.2%) and articular collapse (24.5%) at the knee [16]. This limits our ability to compare our data with those of these studies. Our findings are being supported by the study of Cahir et al.: they assessed multiple regions in sequential scans for regional migratory osteoporosis and found that the ankle and foot and the knee were most affected when the lesions recurred [17].

Previous studies showed that the mean age of disease onset in BMES is 30–60 years [19]. While we also detected a large peak of BMES cases in 51–70-year-olds, we also observed a small peak of BMES cases in adolescents. This interesting observation most likely reflects the fact that pubertal growth and aging both associate with relatively insufficient bone mineralisation that can increase bone fragility [20, 21].

The fact that our BMES cases are prevalent in a state of insufficient bone metabolism and high bone turnover is consistent with observations of this study. First, we found that a relatively large proportion of our patients (12%) were on long-term systemic corticosteroid therapy. This increases bone fragility by promoting bone resorption [22]. Second, a surprisingly large number of our patients (21.5%) had been diagnosed previously with thyroid disorders (almost exclusively hypothyroidism) and altogether 16% were being treated with thyroid hormone at the time BMES was diagnosed. Several other studies reported that the frequencies of thyroid disorders in their BMES patient cohorts ranged from 4 to 19% and that 10% were taking thyroid hormone at BMES diagnosis [23–25]. While hyperthyroidism is well known to induce catabolic bone metabolism, there is also some suspicion that hypothyroidism can promote bone fragility by reducing bone turnover and prolonging the bone remodelling cycle [26]. Moreover, thyroid hormone therapy associates with an increased risk of fracture [26], although the underlying mechanism remains to be elucidated. Third, there was a high frequency of secondary hyperparathyroidism in our cohort (21.4%). In normal populations, 2.7–6.6% have secondary hyperparathyroidism [27, 28]. The high frequency of secondary hyperparathyroidism in our cohort is consistent with the low average calcium serum levels, relatively high PTH levels and average DPD levels that exceeded the upper reference value. Fourth, we found that 5% of our patients had manifest PPI-induced hypochlorhydria, which associates with bone fragility [29]. Fifth, we found reduced trabecular thickness and cortical volumetric BMD. Similar alterations in bone structure were found in patients with primary hyperparathyroidism [30] and may display osteomalacia. All of these findings suggest that patients with BMES have high bone turnover. If this is causal for the development of BMES or a consequence of remains unclear. Berger et al. found that, although patients with BMES had normal serum bone turnover variables (BAP, osteocalcin and C-terminal crosslinking telopeptide), aspirates of the cancellous bone of the BMES lesions had high levels of these markers [9]. Overall, these previous observations are consistent with the fact that anti-resorptive therapy (i.e., bisphosphonates, denosumab) was found to be effective in the treatment of BMES [31–33].

The possibility that BMES associates with impaired calcium metabolism is further supported by our finding that vitamin D insufficiency and deficiency were very common in our cohort (frequencies of 92 and 58% in the patients without and with recent vitamin D supplementation, respectively). Our findings are supported by Horas et al. (*n = 31*) and Sprinchorn et al. (*n = 10*), who also found that patients with BMES frequently had vitamin D insufficiency (defined as < 30 μg/l, 84 and 90%, respectively) and a low average vitamin D level (19 μg/l) [7, 8]. While recent epidemiological data show that these high frequencies of vitamin D insufficiency are not dissimilar to those in the general German population (88% have 20–30 μg/l, 62% have < 20 μg/l and the mean level is 18 μg/l) [34], such extensive vitamin D insufficiency is likely to contribute to the derangement of bone metabolism/bone mineralisation that is seen in BMES.

Notably, given the high prevalence of vitamin D deficiency in our cohort, it is likely that this is largely responsible for the high prevalence of secondary hyperparathyroidism. Other factors such as PPI-induced hypochlorhydria may also contribute. It should be noted that, although 36% of our patients were taking vitamin D supplements at the time BMES was diagnosed (and PTH was measured), this supplementation only started very recently in most of these patients. Consequently, the PTH levels of the cohort patients had not yet had the time to respond to the supplementation in most cases.

BMES lesions were originally called transient osteoporosis. However, this name was dropped because DXA studies showed that patients with bone marrow lesions do not have always decreased BMD [8, 15, 16]. This is supported by the present study. Although we found that 47% of our patients had osteopenia and 18% had osteoporosis, several studies reported that up to 15% of the general population have osteoporosis [35, 36]. Thus, our cohort did exhibit marginal reduced BMD compared to the general population. Although metabolism derangement does appear to associate strongly with BMES, the pathogenesis of this disease does not seem to require substantial alterations in bone mineral density as reflected by DXA.

To sum up, this study showed that BMES lesions, which almost exclusively affect weight-bearing and thus load-affected joints, may be caused by impaired bone metabolism during adolescence and older age that is the result of high systemic bone turnover, as possible consequence of reduced vitamin D levels. We thus hypothesise that BMES pathogenesis is a multifactorial process in which local or systemic phases of accelerated bone turnover cause bone microdamage, even under physiological load, and that this bone microdamage may predispose the individual to the development of BMES. This hypothesis is entirely consistent with the increasingly accepted pathophysiology-based concept, namely, that diseases such as BMES may be “regional acceleratory phenomena” where a noxious stimulus such as bone tissue microdamage in vitamin D deficient bone causes normal biological processes, including blood flow, cell metabolism and tissue remodelling processes, to accelerate. This in turn leads to a transient shift to unmineralised bone [37]. This possibility is supported by histological and biochemical findings of BME lesion specimens [5, 9, 10]. Thus, BMES may be the result of decoupling between the self-repairing capacity of the bone tissue and the accumulation of bone microdamage due to impaired bone metabolism [4].

This study has a number of limitations. First, its cross-sectional design makes it difficult to identify a causal association between BMES and impaired calcium metabolism and bone mineralisation. Second, the data are retrospective, which introduces the possibility of information bias. Third, there may have been some selection bias, which may limit the generalisability of our findings to other BMES populations. However, these limitations are unlikely to affect the main conclusions of the study.

## Conclusions

We conclude that patients diagnosed with primary bone marrow lesions should be screened carefully for bone metabolism disorders, especially vitamin D levels, and their potential risk factors. Moreover, our findings suggest that a comprehensible treatment for BMES is a therapy that compensates for elevated bone resorption.

## Additional file

Additional file 1: **Figure S1.** Distribution of bone marrow oedema lesions during winter and summer time (82/98 bone marrow oedema lesions). (TIF 17 kb)

## Abbreviations

25-OH-D_3_: 25-hydroxyvitamin D
BAP: Bone specific alkaline phosphatase
BMD: Bone mineral density
BMES: Bone marrow oedema syndrome
DPD: Deoxypyridinoline
DXA: Dual-energy X-ray absorptiometry
HR-pQCT: High-resolution peripheral quantitative computed tomography
MRI: Magnetic resonance imaging
PPI: Proton pump inhibitor
PTH: Parathyroid hormone
