# Supplementary figures and images for: Identification of vitamin D and other bone metabolism parameters as risk factors for primary bone marrow oedema syndrome
Source: BMC Musculoskelet Disord. 2018 Dec 22;19:451. doi: 10.1186/s12891-018-2379-x (PMC6303903; doi:10.1186/s12891-018-2379-x)

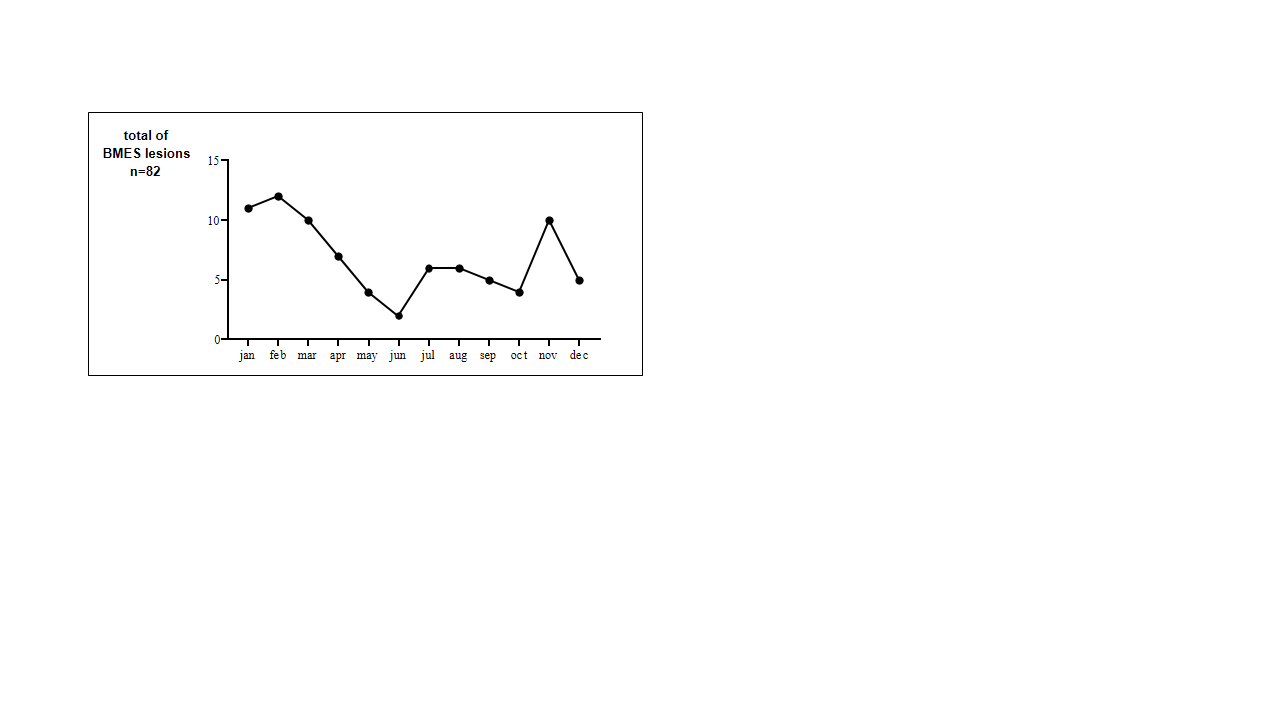

Supplement: Supplementary file 1 — Figure S1. Distribution of bone marrow oedema lesions during winter and summer time (82/98 bone marrow oedema lesions). (TIF 17 kb) [file 12891_2018_2379_MOESM1_ESM.tif]
